# Artificial intelligence and automation in enzyme engineering: evolution, advances, and future perspectives

**DOI:** 10.1186/s40643-026-01096-3

**Published:** 2026-07-10

**Authors:** Kexin Hao, Jianguang Liu, Hui Tang, Yan Zhang, Yandong Sun, Hongyu Zhang, Ji Wang, Peng Liu, Jianmei Luo, Jing Zhao

**Affiliations:** 1 State Key Laboratory of Druggability Evaluation and Systematic Translational Medicine, Tianjin Institute of Pharmaceutical Research, 306 Huiren Road, Tianjin, 300301 China; 2 State Key Laboratory of Bio-based Fiber Materials, Key Laboratory of Industrial Fermentation Microbiology (Tianjin University of Science &Technology), Tianjin Key Laboratory of Industrial Microbiology, Tianjin Engineering Research Center of Microbial Metabolism and Fermentation Process Control, College of Biotechnology, Ministry of Education, Tianjin University of Science and Technology, Tianjin, 300457 China; 3 Beijing MegaRobo Technologies Co., Ltd, 901, 9th Floor, Block A, Longyu Center, No. 1 Yard, Longyuzhongjie, Huilongguan, Changping District, Beijing, 100085 China

**Keywords:** Enzyme engineering, Artificial intelligence, Automation technologies, Protein language models, Biofoundry, Design-build-test-learn (DBTL) cycle

## Abstract

Natural enzymes often fail to meet industrial demands for catalytic efficiency, stability, and substrate specificity, creating a critical bottleneck in biomanufacturing. This review examines how artificial intelligence (AI) and automation are reshaping enzyme engineering from empirical trial‑and‑error toward data-driven, closed-loop design. We trace AI development from feature-engineered machine learning to supervised deep learning and self-supervised protein language models, and automation from standalone task execution to cascade integration and biofoundry-enabled build-test workflows. Their convergence is analyzed through a stage-based autonomy framework, highlighting the transition from semi-automated workflows to conditional and high-autonomy DBTL systems. Recent studies demonstrate that AI-guided prediction, automated experimentation, and active learning can accelerate enzyme optimization; however, key barriers remain, including biased datasets, limited out-of-distribution generalization, weak mechanistic interpretability, automation interoperability constraints, and unresolved multi-objective trade-offs. We discuss future directions involving FAIR-compliant data infrastructure, hybrid sequence-structure-physics models, modular automation platforms, and autonomous closed-loop systems. By integrating historical evolution, representative case studies, success and failure analysis, and practical bottlenecks, this review provides a roadmap for advancing AI-guided and autonomous enzyme engineering.

**Supplementary Information:**

The online version contains supplementary material available at 10.1186/s40643-026-01096-3.

## Introduction

Biomanufacturing has emerged as a strategically important industrial sector, providing a transformative route to achieve efficient and green production of high-value compounds through either biocatalysis or cell factories (Asin-Garcia et al. 2025; Shi et al. 2022; Yan et al. 2024). Enzymes are central functional components of biomanufacturing systems, where they catalyze biological reactions involved in the production of high-value chemicals (Farhan et al. 2025; Reetz et al. 2024; Wu et al. 2020). However, natural enzymes often fail to meet industrial demands in terms of catalytic efficiency, stability, and substrate specificity, creating a critical bottleneck that limits the large-scale development of biomanufacturing (Buller et al. 2023). Therefore, developing engineered enzymes that are efficient, stable, and well-adapted to industrial processes is of strategic importance for overcoming technological bottlenecks and promoting high-quality development in the biomanufacturing sector (Ndochinwa et al. 2024).

Traditional enzyme engineering techniques have primarily relied on directed evolution and rational design (Chowdhury and Maranas 2019; Ferreira et al. 2022). These approaches optimize enzyme performance by constructing mutant libraries coupled with high-throughput screening (Xiao et al. 2014), or by guiding amino acid substitutions at key sites based on protein structural information (Phintha and Chaiyen 2022). However, both methods have inherent limitations: directed evolution faces challenges related to the massive scale of mutant libraries, limited screening throughput, and lengthy cycles (Wang et al. 2021); rational design is highly dependent on the availability of high-resolution structural information and is constrained by an incomplete understanding of enzyme structure–function relationships (Qin et al. 2024). In recent years, AI with its powerful data mining and predictive capabilities, has enabled increasingly reliable mapping from enzyme sequences to functional properties, offering a new “intelligent design” paradigm for enzyme engineering (Lu et al. 2025; Shi et al. 2026a; Yang et al. 2025a, b). Concurrently, the rapid development of automated equipment and biofoundries has enabled the build and test stages of the DBTL cycle to operate efficiently under unattended conditions, greatly improving experimental throughput and data quality (Dixit et al. 2021; Giuseppe et al. 2026; Yu et al. 2023b). The deep integration of AI and automation technologies is driving a substantial transformation in enzyme engineering (Chen et al. 2025; Orouji et al. 2025). It shifts the field from empirical trial-and-error to data-driven approaches, and from labor-intensive to intelligent and efficient workflows, with the potential to improve design efficiency and iteration speed in enzyme engineering (Chen et al. 2025).

This review provides a critical and integrated analysis of the evolution, current advances, and future directions of AI- and automation-enabled enzyme engineering. Unlike reviews that primarily summarize individual computational methods or automated platforms, we trace the parallel development of AI models and laboratory automation, highlight representative enzyme-engineering cases, and analyze the factors underlying both successful and less successful applications. More importantly, we distinguish AI-guided enzyme engineering from autonomous enzyme engineering and critically examine the major barriers that currently limit their broader implementation, including data quality, benchmark reliability, out-of-distribution generalization, mechanistic interpretability, epistasis, multi-objective optimization, build-test bottlenecks, platform interoperability, and autonomy gaps. By integrating technological evolution, case-based analysis, bottleneck diagnosis, and potential solutions, this review aims to provide a practical roadmap for applying AI and automation to enzyme engineering and to inform future efforts toward closed-loop and autonomous enzyme optimization.

## AI and automation in enzyme engineering: evolution and milestones

**Fig. 1:**
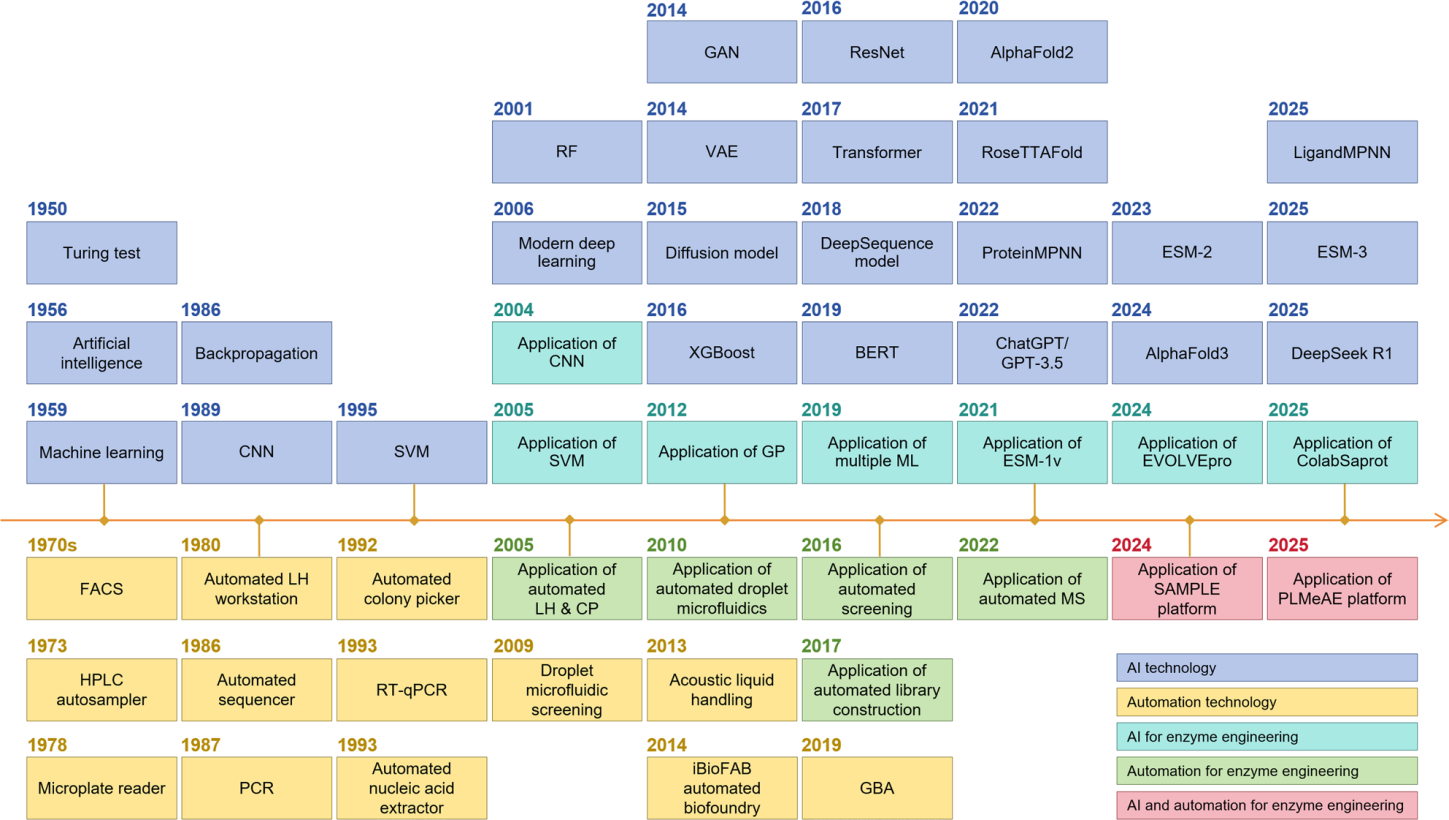
Timeline of AI and automation technologies in enzyme engineering. Abbreviations: CNN, Convolutional Neural Network; CP, Colony Picking. FACS, Fluorescence-Activated Cell Sorter; GAN, Generative Adversarial Network; GBA, Global Biofoundry Alliance; GP, Gaussian Process; HPLC, High Performance Liquid Chromatography; LH, Liquid Handling; ML, Machine Learning; RF, Random Forest; SVM, Support Vector Machine; VAE, Variational Autoencoder; XGBoost, Extreme Gradient Boosting

This section systematically reviews the development trajectory and key milestones of AI technology, automation technology, and their convergence in the field of enzyme engineering (Singh et al. 2025). Figure 1 presents a timeline illustrating the evolution of core AI algorithms and their milestone applications in enzyme engineering, the technological leap of automation equipment from standalone operation to increasingly integrated workflows, and representative achievements enabled by the deep integration of AI and automation in recent years (Kwon 2024). Together, these elements outline a clear evolutionary trajectory of the field from empirical trial-and-error toward data-driven intelligent design.

### Evolution of AI in enzyme engineering

#### Three major stages of AI development categorized by learning paradigm

The development of AI can be broadly divided into three major stages according to the dominant learning paradigm, reflecting how AI systems extract and generalize knowledge from data (Oh 2019; Vaswani et al. 2017). The first stage was dominated by rule-based reasoning and feature-engineered machine learning (ML), in which models relied heavily on handcrafted descriptors and domain-specific knowledge. The second stage was characterized by supervised deep representation learning, where deep neural networks enabled automatic feature extraction from large labeled datasets. The third and current stage is driven by large-scale self-supervised foundation models, represented by Transformer-based architectures, which learn transferable representations from massive unlabeled datasets. In protein engineering, this paradigm has given rise to protein language models (PLMs), which learn evolutionary and functional representations from large-scale protein sequence corpora.

#### Three-stage application of AI in enzyme engineering

AI applications in enzyme engineering have evolved alongside advances in AI methodologies, progressing through three overlapping stages: feature-engineered ML, supervised deep representation learning, and large-scale self-supervised foundation models. These stages reflect the increasing ability of AI models to represent enzyme sequence–structure–function relationships, prioritize beneficial mutations, and support closed-loop optimization. Importantly, these stages are not strictly sequential in practice; conventional ML models such as random forests (RFs) and support vector machine (SVM)-based methods remain widely used, especially when datasets are small, interpretable features are available, or task-specific experimental data are limited.

Feature-engineered ML stage (emerging in the early 2000s). In this stage, AI models primarily relied on handcrafted sequence- or structure-derived descriptors combined with conventional ML algorithms such as RFs, SVMs, and shallow neural networks. These methods typically focused on specific predictive tasks, such as estimating mutation effects on protein stability or activity. An early representative example was I-Mutant (2004), a neural network-based method that predicted the direction of single-point mutation effects on protein stability (ΔΔG sign) using protein structural information (Capriotti et al. 2004). Subsequently, I-Mutant2.0 (2005) introduced SVM-based regression to predict quantitative ΔΔG values and enabled stability prediction directly from sequence, representing one of the earliest practical AI tools for enzyme engineering (Capriotti et al. 2005; Khan and Khan 2025). Although limited by small datasets and manually designed features, these early methods established the foundation for AI-assisted enzyme optimization.

Supervised deep representation learning stage (gaining momentum in the 2010s). This stage marked the transition from feature-engineered ML to end-to-end deep neural networks capable of learning hierarchical sequence–structure–function representations directly from data. Compared with traditional ML, deep learning (DL) reduced reliance on handcrafted descriptors and enabled automatic feature extraction, substantially improving predictive performance for complex protein engineering tasks. During this period, DL architectures such as Convolutional Neural Networks (CNNs) and Recurrent Neural Networks (RNNs), and fully connected Deep Neural Networks (DNNs) were increasingly applied to enzyme engineering. In 2018, DeepSequence employed a variational autoencoder (VAE)-based deep generative model to learn evolutionary constraints from multiple sequence alignments, introducing unsupervised representation learning for mutation effect prediction and marking an important shift toward data-driven sequence modeling (Riesselman et al. 2018). In 2019, DeepDDG employed a three-layer feedforward neural network to predict mutation-induced stability changes (ΔΔG) using sequence- and structure-derived features, further demonstrating the utility of DL for quantitative enzyme property prediction (Cao et al. 2019).

Large-scale self-supervised foundation model stage (emerging in the early 2020s and ongoing). This stage is characterized by large-scale self-supervised pretraining on protein sequence and structure datasets, enabling transfer learning, zero-shot prediction, generative design, and closed-loop optimization. These capabilities improve generalization across diverse enzyme families and engineering tasks. A major milestone during this period was AlphaFold2 (2020), which dramatically advanced high-accuracy protein structure prediction and accelerated the transition from sequence-based engineering toward structure-informed enzyme design (Jumper et al. 2020). In 2021, the ESM series of PLMs enabled zero-shot prediction of mutation effects directly from large-scale sequence pretraining (Meier et al. 2021). More recently, AI-guided enzyme engineering has increasingly been coupled with active learning and laboratory automation. In 2024, the EVOLVEpro framework combined a PLM with active learning to achieve rapid directed evolution through an iterative “predict–test–learn” closed loop, which represents an important step in active learning-driven closed-loop optimization (Jiang et al. 2024a, b). The current stage extends beyond PLMs to include structure-aware geometric DL, generative AI, and automated experimentation, shifting enzyme engineering from predictive modeling toward autonomous optimization.

### Evolution of automation in enzyme engineering

#### Hardware foundations: from industrial robotics to biofoundries

The development of automation technology is rooted in advances in machinery, electronics, and computer science. From the late 1950s to the early 1960s, the advent of industrial robotic arms marked the era of automated physical manipulation. In the 1970s, the development of microprocessors enabled the automation of stand-alone equipment. The introduction of the Multiskan microplate reader in 1978, the launch of fully automated liquid handling workstations in 1980, and the release of the commercialized polymerase chain reaction (PCR) instrument TC1 in 1987 signaled the arrival of stand-alone automation in the life sciences. In the 1990s, the application of automated colony pickers effectively addressed throughput bottlenecks in microbial cloning. After 2000, advances in robotics, microfluidic control, and system integration led to the maturation of technologies such as flow cytometers and integrated liquid handling workstations. In the 2010s, breakthroughs in droplet microfluidics enabled high-throughput analysis of picoliter-scale reactions (Brouzes 2009). In recent years, driven by the principles of Industry 4.0 and Laboratory 4.0, emerging technologies such as integrated biofoundries and acoustic droplet ejection have continued to emerge, establishing the hardware foundation for full automation in enzyme engineering.

#### Three stages of automation‑driven enzyme engineering

Against the backdrop of continuous advances in automation equipment, the application of automation technology in enzyme engineering (excluding AI-driven decision-making) has evolved through three developmental stages: from standalone task automation, through cascade workflow integration, to highly automated build–test workflows.

Standalone automation stage (emerging in the 2000s) primarily employed automated colony pickers, liquid handling workstations, and fluorescence-activated cell sorting (FACS) to replace specific, labor‑intensive manual steps. For example, Aharoni et al. (2006) used FACS to sort a sialyltransferase mutant library (Aharoni et al. 2006), while Lipovsek et al. (2007) combined yeast surface display with FACS to screen horseradish peroxidase libraries (Kwon 2024; Lipovšek et al. 2007). These early efforts demonstrated that single automated instruments could dramatically accelerate the testing phase of enzyme engineering.

Cascade automation stage (gaining momentum in the 2010s) was characterized by the logical serial integration of multiple standalone devices via robotic arms and unified software protocols. A representative work by Chen et al. (2011) combined yeast surface display, FACS, and robotic liquid handling to evolve sortase A (Chen et al. 2011). Similarly, Ostafe et al. (2014) integrated automated colony picking, liquid handling, and microplate reading to screen a glucose oxidase library (Ostafe et al. 2014). Dörr et al. (2016) established a fully automated robotic platform that orchestrated colony picking, cell cultivation, protein expression, cell lysis, and activity assays for high-throughput screening of diverse enzyme classes (Dörr et al. 2016). These systems reduced manual intervention and improved data reproducibility by semi‑automating the transition between growth, induction, and activity measurement.

Highly automated build–test stage (emerging in the early 2020s and ongoing) is marked by deep automation coupling of the “build–test” cycles, often within integrated biofoundries. Zhang et al. (2022) developed an end‑to‑end robotic workflow incorporating automated library construction, microplate cultivation, and MALDI‑ToF MS screening for directed evolution of a cyclodipeptide synthase (Zhang et al. 2022). These platforms now routinely handle thousands of variants per day with minimal human intervention, driving a paradigm shift toward highly automated “build–test” execution. In recent years, an increasing number of studies have integrated automation with AI for enzyme engineering, moving beyond the sole use of automation to gradually achieve a complete DBTL cycle (Heo et al. 2025).

### Evolution of integrated AI–automation systems in enzyme engineering

The deep integration of AI and automation technologies is driving a paradigm shift in enzyme engineering, moving from “modular execution” toward “autonomous iteration”. To distinguish workflow automation from decision-making autonomy, we adopt a six-level autonomy framework (Levels 0–5) for enzyme engineering workflows, adapted from recent laboratory automation frameworks (Beal and Rogers 2020; Singh et al. 2025). Level 0 represents fully manual operation, while Levels 1–2 correspond to increasing degrees of task and workflow automation with substantial human oversight. Level 3 marks conditional autonomy, where systems can execute multiple DBTL cycles under predefined conditions with limited human intervention. Level 4 represents high autonomy with minimal human intervention, whereas Level 5 corresponds to full autonomy, where end-to-end DBTL cycles are executed without human involvement. Since the early 2020s, the field has been transitioning from lower autonomy (Levels 1–2) toward conditional and high autonomy (Levels 3–4), while Level 5 remains the long-term frontier (Singh et al. 2025).

The lower-autonomy stage (Levels 1–2) represents the initial integration phase. ML models were preliminarily coupled with automated liquid handling workstations, enabling partially automated linkages in the “design–build–test” cycle. However, critical steps such as library construction, model training, or data curation still required manual intervention, and end-to-end closed-loop operation was not yet achieved. A representative study from this level is the systematic engineering of artificial metalloenzymes for bioorthogonal reactions using regression models coupled with automated liquid handling (Vornholt et al. 2021). This early effort established the foundational workflow architecture for ML-guided enzyme engineering but remained fundamentally human-dependent across iterative cycles.

**Fig. 2:**
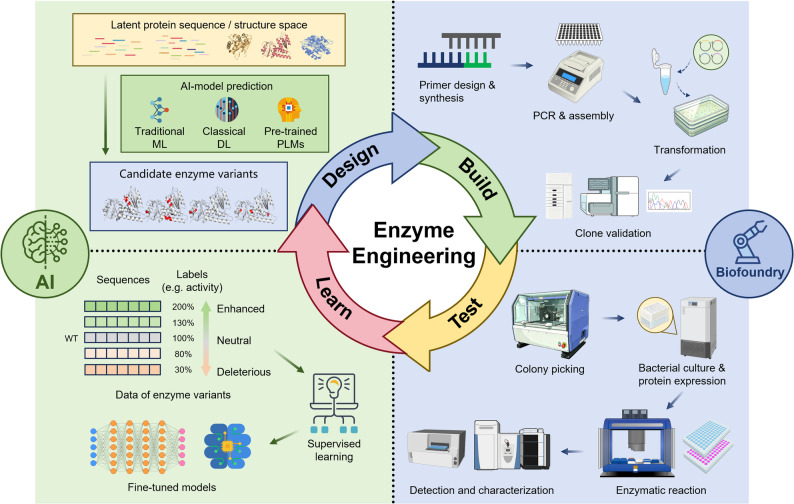
The DBTL (Design–Build–Test–Learn) cycle in enzyme engineering, powered by AI and automation technologies. Design: Leveraging latent protein sequence/structure space through AI-model prediction, traditional ML, classical DL, and pre-trained protein language models (PLMs). Build: Automated workflows including primer design & synthesis, PCR & assembly, transformation and clone validation. Test: Colony picking, high-throughput bacterial culture & protein expression, followed by detection, characterization, and enzymatic reaction assays. Learn: Supervised learning and fine-tuned models using data from enzyme variants to iteratively improve predictions and guide subsequent design cycles

Current state-of-the-art platforms are progressing toward higher autonomy through the integration of PLMs, active learning, and biofoundries. At this stage, AI models increasingly guide automated experimentation across multiple DBTL cycles (Fig. 2), significantly reducing human intervention (Yu et al. 2023a). Representative advances include a generalized platform integrating PLMs and supervised learning with a biofoundry to enhance enzyme catalytic activity and substrate preference (Singh et al. 2025), and a PLM-enabled automated evolution system coupled with automated mutagenesis and screening to improve non-canonical amino acid incorporation efficiency (Zhang et al. 2025b). Notably, Singh et al. (2025) explicitly characterized their system as operating between Level 3 and Level 4, highlighting that even the most advanced platforms have not yet achieved full autonomy. Nevertheless, despite substantial progress, current systems still require limited human involvement for initialization, error handling, and experimental validation, indicating that fully autonomous DBTL operation remains unrealized.

The fully autonomous closed-loop level (Level 5) represents the future frontier, where end-to-end DBTL cycles will operate without human intervention, including initialization, optimization, error recovery, and decision-making. The progression toward this level is being driven by advances in LLM-based multi-agent systems and increasingly robust hardware-software integration. Collectively, the synergy between AI and automation is steadily evolving toward higher-level autonomous closed-loop systems and broader platform generalizability.

## Applications of AI and/or automation in enzyme engineering

### AI-driven enzyme engineering

AI has become a pivotal force in enhancing enzyme performance, with applications ranging from predicting enzyme reaction parameters to guiding complex functional optimization. Current AI-driven enzyme engineering methods fall into two broad categories based on their reliance on experimental data from the target enzyme (Liu et al. 2025): (i) zero-shot prediction methods, which leverage pre-trained models (e.g., ESM-1v) trained on massive public datasets and require no experimental data from the target enzyme to forecast mutation effects; and (ii) supervised learning methods, which require experimental data from the target enzyme or its homologs (e.g., mutant activities) to train or fine-tune models. By combining traditional ML, DL, and emerging structure-aware or generative models, researchers can more efficiently explore large sequence spaces and optimize enzymes across diverse families, including oxidoreductases, transferases, hydrolases, lyases, and isomerases. Table 1 summarizes representative examples since 2020 of AI applications in enzyme engineering, illustrating the breadth and potential of AI-guided optimization across major enzyme classes.

**Table 1:** Representative cases of AI-driven enzyme engineering

No.	EC classes	Enzyme	AI tools or algorithms	Prediction type	Enzyme engineering result	Reference
1	EC 1 Oxidoreductases	*Sporidiobolus salmonicolor* ketoreductases (Ssal-KRED)	Gaussian process	Supervised learning	64-fold higher *k*_cat_	(Honda Malca et al. 2024)
2	EC 1 Oxidoreductases	*Pseudomonas aeruginosa* p-hydroxybenzoate hydroxylase (PHBH)	SVR with Evotuned-BERT features	Supervised learning	> 100-fold higher activity	(Matsushita et al. 2024)
3	EC 1 Oxidoreductases	*Candida albicans* ene reductase (ERED)	Linear SVR	Supervised learning	Measured *T*_m_ (58 °C) matched the predicted Δ*T*_m_ of + 5 °C	(Brennan et al. 2024)
4	EC 1 Oxidoreductases	*Sphingobium sp.* CSO carotenoid cleavage oxygenase (SsCSO)	CataPro	Supervised learning	~65-fold higher activity	(Wang et al. 2025a, b)
5	EC 2 Transferases	*Thermocrispum agreste* UDP-glucose pyrophosphorylase (TaUGP)	ProteinMPNN; ancestral sequence reconstruction	Zero-shot prediction	~ 500-fold greater stability	(Li et al. 2024b)
6	EC 2 Transferases	*Ruegeria sp.* TM1040 (S)-selective transaminase (3FCR)	GBRT	Supervised learning	Up to 3-fold higher activity for bulky substrates and > 99% ee	(Menke et al. 2024)
7	EC 2 Transferases	T7 RNA polymerase	Pro-PRIME	Zero-shot prediction	3.5 °C higher *T*_m_ and 40-fold higher activity after heat shock	(Jiang et al. 2024a)
8	EC 3 Hydrolases	*Rhodococcus erythropolis* AJ270 nitrile hydratase/amidase	Random forest classification	Supervised learning	53-fold higher *E*-value	(Li et al. 2024a)
9	EC 3 Hydrolases	*Bacillus licheniformis* endonuclease (NucB)	TeleProt	Supervised learning	19-fold higher specific activity at pH 7	(Thomas et al. 2025)
10	EC 3 Hydrolases	Polyethylene terephthalate hydrolase (PES-H1)	iCASE	Supervised learning	11.09-fold activity and 7.5 °C higher *T*_m_	(Zheng et al. 2025)
11	EC 4 Lyases	*Rhodotorula glutinis* tyrosine ammonia lyase (RgTAL)	UniKP	Supervised learning	3.5-fold higher *k*_cat_/*K*_m_	(Yu et al. 2023a)
12	EC 4 Lyases	*Bacillus licheniformis* pectin lyase PMGL-Ba	SVR	Supervised learning	67-fold (75℃) and 39-fold (80℃) longer in half-life and 2.1-fold higher activity	(Zhang et al. 2024)
13	EC 4 Lyases	*E. coli* hexamer glutamate decarboxylase (GADA)	iCASE	Supervised learning	2.34-fold higher specific activity and 2.0 °C higher *T*_m_	(Zheng et al. 2025)
14	EC 5Isomerases	*Candida albicans *phosphomannose isomerase (CaPMI)	3D convolutional neural network	Zero-shot prediction	5-fold more fluorescent	(Shroff et al. 2020)
15	EC 5Isomerases	*Comamonas testosterone* ketosteroid isomerase (KSI)	AI.zymes	Supervised learning	7.7-fold higher *k*_cat_/*K*_m_ and remained folded up to 95 °C	(Merlicek et al. 2025)
16	EC 6Ligases	*Marinactinospora thermotolerans* ATP-dependent amide bond synthetase (McbA)	Augmented ridge regression	Supervised learning	42-fold higher catalytic efficiency and 96% conversion	(Landwehr et al. 2025)
17	EC 6Ligases	*Methanosarcina mazei* pyrrolysyl-tRNA synthetase (PylRS)	FFT-PLSR; ESM-1v; MutCompute；ProRefiner	Hybrid strategy	30.8-fold higher SCS efficiency and up to 7.8-fold higher catalytic efficiency	(Zhang et al. 2025b)

*SVR *Support Vector Regression, *GBRT *Gradient Boosting Regression Tree, *iCASE *Isothermal Compressibility-assisted Dynamic Squeezing Index Perturbation Engineering, *FFT-PLSR *Fast Fourier Transform-Partial Least Squares Regression

For oxidoreductases, AI has demonstrated promising capacity to enhance both catalytic activity and thermostability. Brennan et al. combined ancestral sequence reconstruction with ML to engineer an ene reductase (Brennan et al. 2024). By constructing a combinatorial mutant library based on the uncertainty in the amino acid probability distributions calculated during the ancestral sequence reconstruction of ER120, they trained a linear support vector regression (SVR) model using screening data covering only 0.35% of the library, successfully predicting the optimal variant ER218, which exhibited a melting temperature (*T*_m_) 5 °C higher than its parent ER120 and superior conversion across multiple substrates. A plausible explanation for this outcome is that ASR-derived uncertainty appeared to enrich thermostabilizing mutations, as evidenced by the improved *T*_m_ observed in 11 of 17 single-site variants. In addition, the thermostability landscape appeared relatively smooth, with limited higher-order epistasis, allowing effective generalization from sparse sampling. In contrast, catalytic activity remained difficult to predict, likely due to more complex sequence-function relationships and limited activity variation. Table 1 summarizes representative studies demonstrating the application of AI across different enzyme-engineering tasks. Although these studies collectively highlight the growing impact of AI-guided approaches, direct comparisons across studies should be interpreted cautiously, as reported performance can be strongly influenced by differences in datasets, engineering objectives, assay conditions, screening strategies, and evaluation metrics.

In transferase engineering, AI has shown substantial promise in extracting insights from limited experimental data to efficiently explore complex combinatorial spaces and improve the conversion of non-natural substrates. For UDP-glucose pyrophosphorylase (TaUGP), Li et al. adopted a strategy combining the DL method ProteinMPNN with ancestral sequence reconstruction to screen key mutation sites (Li et al. 2024b). The triple mutant M-opt, generated through combinatorial mutagenesis, exhibited a 500‑fold improvement in thermostability at 60 °C compared to the wild-type, with a half‑life of 49.8 h, thus laying a solid foundation for the in vitro multi-enzymatic cascade synthesis of UDP-glucose. The favorable outcome may be attributable to the constrained design strategy, in which catalytic residues and the active-site microenvironment were preserved. This allowed AI-guided mutations to improve structural stability while minimizing disruption of catalytic function. The strong additivity among the selected stabilizing mutations also reduced epistatic complexity, making combinatorial optimization more predictable.

In the field of hydrolases, AI has shown potential to address the long-standing engineering bottleneck of simultaneously improving stability and activity, and has enabled effective prediction and tuning of enantioselectivity. For PET hydrolase (PES-H1), Zheng et al. proposed the iCASE strategy, integrating dynamic structural features such as isothermal compressibility and the dynamic squeezing index to build a RF regression model (Zheng et al. 2025). The predicted and experimentally validated variant T63H exhibited an 11.09‑fold increase in enzymatic activity and a 7.5 °C increase in *T*_m_, achieving a synergistic enhancement of thermostability and catalytic activity. The observed improvement suggests that incorporating conformational dynamics provides information that is not fully captured by conventional static-structure descriptors. However, its predictive power for activity varies considerably across enzyme families, and extrapolation to higher-order mutants remains sensitive to the degree of epistatic contribution in the fitness landscape.

In lyase and isomerase engineering, AI has also successfully addressed the “combinatorial explosion” challenge and achieved synergistic improvements in thermostability and catalytic efficiency. In a study engineering the Kemp eliminase activity of ketosteroid isomerase (KSI), Merlicek et al. developed the modular AI.zymes platform, integrating Rosetta, ProteinMPNN, ESMFold, and FieldTools within an evolutionary design framework (Merlicek et al. 2025). Using multi‑objective Boltzmann selection to simultaneously optimize transition‑state recognition, protein stability, and catalytic electric fields, and after multiple rounds of iterative design and testing of only seven variants, they obtained the optimal variant K3. This variant exhibited a 7.7‑fold increase in the maximum *k*_cat_/*K*_m_ compared to the wild-type, with *T*_m_ increased from 49.8 °C to over 95 °C, powerfully illustrating the potential value of AI in multi‑objective co‑optimization. These results suggest that evolutionary multi-objective selection can effectively optimize properties beyond those explicitly encoded by individual design algorithms, provided that such properties can be quantitatively evaluated. This capability partially mitigates the stability-centric biases of widely used design tools such as Rosetta and ProteinMPNN.

Taken together, these representative cases demonstrate the substantial potential of AI to accelerate mutation prioritization, combinatorial-space reduction, and functional optimization across diverse enzyme classes. However, direct cross-study comparison remains difficult because performance improvements are reported using heterogeneous metrics, experimental conditions, and evaluation criteria. Moreover, published studies are strongly enriched for successful applications, making it difficult to assess the true robustness and generalizability of current AI approaches from success stories alone. Importantly, the current literature is also affected by a strong publication bias toward successful engineering campaigns. As a result, reported benchmarks and case studies may not accurately reflect real-world success rates, and the practical limitations of AI-guided enzyme engineering remain difficult to quantify. Therefore, comparative analysis of both successful and failed applications is necessary to define the practical boundaries of current AI methods.

### Determinants of success and failure in AI-guided enzyme engineering

Building on the representative case studies above, it becomes clear that AI performance in enzyme engineering is highly context dependent. Successful engineering campaigns rarely rely on AI alone. Instead, they typically combine AI with complementary approaches such as ancestral sequence reconstruction, structural modeling, active learning, or automated experimentation (Landwehr et al. 2025; Liu et al. 2024). Therefore, the utility of AI should be evaluated not simply by isolated success stories, but by how well model assumptions align with the underlying engineering task. This section analyzes AI performance from two complementary perspectives: (a) comparative evaluation of the same AI tool across successful and failed applications; and (b) comparative assessment of different AI tools applied to the same engineering task.

Comparison of the same AI model across different application contexts shows that success is highly conditional on task compatibility. For example, supervised kinetic predictors such as DLKcat can be useful when target enzymes remain close to the training distribution and the engineering objective is limited to interpolation, such as prioritizing single-site mutations in homologous proteins (Liu et al. 2023). However, their performance deteriorates sharply under out-of-distribution conditions, including distant homologs, non-native substrates, and multi-site combinatorial mutations (Kroll A and M. 2024; Shao et al. 2025). These failures often arise from benchmark leakage, strong sequence redundancy in training datasets, and poor modeling of epistatic interactions. In contrast, zero-shot PLMs such as ESM-1v generally show better robustness across moderately out-of-distribution sequence variants because they capture broad evolutionary constraints (Zeng et al. 2026). Nevertheless, their predictive power remains limited when catalytic performance depends strongly on structural geometry, substrate recognition, or higher-order residue interactions, as demonstrated in engineering tasks involving substrate-specific or structurally constrained active sites (Zhang et al. 2025a). In practice, such models are most effective for mutation prioritization rather than autonomous enzyme design.

Comparisons between different AI models applied to the same engineering task further reveal that no single model is universally optimal. Instead, model performance depends strongly on the target property being optimized (Michael et al. 2024; Zhang et al. 2025a). Structure-aware models often perform better in stability-related tasks because thermostability is closely linked to structural determinants such as residue packing, hydrogen-bonding networks, and conformational rigidity. By contrast, sequence-based evolutionary models often perform well for activity prediction because catalytic constraints are partially encoded in sequence conservation and co-evolutionary signals (Zhang et al. 2025a). However, both model classes frequently struggle in selectivity engineering, especially enantioselectivity or substrate specificity, where performance is often substantially lower. This limitation likely reflects the fact that stereoselectivity depends on subtle enzyme-substrate recognition and dynamic conformational effects that are insufficiently captured by current sequence- or structure-based representations.

Taken together, successful AI applications in enzyme engineering consistently share several enabling conditions: high similarity between training and target sequence space, relatively smooth fitness landscapes with limited epistasis, and target properties that correlate with accessible evolutionary or structural signals ( Michael et al. 2024; Zeng et al. 2026). Conversely, failure becomes more likely when these assumptions are violated, particularly during extrapolation to distant homologs, engineering of non-natural substrates, multi-mutation optimization, or tasks that require mechanistic reasoning beyond statistical pattern recognition (Kroll A and M. 2024; Shao et al. 2025). These observations suggest that current AI models should be viewed primarily as search-space reduction tools rather than fully autonomous design engines. Their practical value depends less on benchmark leaderboards and more on careful alignment between model assumptions and the specific engineering problem.

### Automation-driven enzyme engineering

To systematically illustrate the evolutionary trajectory of automation technologies from standalone assistance to highly automated operation and their efficacy in enhancing enzyme engineering efficiency, Table 2 summarizes representative cases of automation-driven enzyme engineering across different developmental stages.

**Table 2:** Representative cases of automation-driven enzyme engineering

No.	Enzyme	Automated process	Screening throughput	Enzyme engineering result	Reference
1	*Campylobacter jejuni* sialyltransferase (CstII)	Automated FACS	~ 10⁶ clones	Catalytic efficiency increased ~ 400-fold	(Aharoni et al. 2006)
2	*Armoracia rusticana* horseradish peroxidase (HRP)	Automated FACS	~ 10⁶ clones	Enantioselectivity enhanced ~ 8-fold	(Lipovšek et al. 2007)
3	*Bacillus sphaericus* phenylalanine dehydrogenase (PheDH)	Automated colony picking; Automated liquid handling workstation	10,000 clones	Catalytic efficiency increased 7.4-fold; Selectivity increased 612-fold	(Chen and Engel 2009)
4	*Campylobacter jejuni* β-1,3-galactosyltransferase (CgtB)	Automated FACS	~ 10⁷ cells/h (sorting rate)	~ 300-fold increased activity	(Yang et al. 2010)
5	*Armoracia rusticana* horseradish peroxidase (HRP)	Droplet-based microfluidics platform	> 2000 droplets/s; ~10⁸ reactions in 10 h	Up to 12-fold higher catalytic activity	(Agresti et al. 2010)
6	*Staphylococcus aureus* sortase A (srtA)	Automated FACS	10⁸ cells per round	140-fold increase in LPETG coupling activity	(Chen et al. 2011)
7	*Acinetobacter* sp. cyclohexanone monooxygenase (CHMO)	Automated robotic platform (colony picker, liquid handler, et al.)	~ 4,200 clones	Identified 6 mutants with reduced product inhibition	(Dörr et al. 2016)
8	*Rhodococcus* sp. M4 phenylalanine dehydrogenase (PheDH)	Automated droplet microfluidics	~ 5 × 10⁵ clones (round 1); ~10⁵ clones (round 2)	Activity increased 4.5-fold; Thermostability increased by 12 °C	(Gielen et al. 2016)
9	*Aspergillus niger* endo-β-1,4-xylanase C (XlnC)	Droplet-based microfluidic HTS platform	10^5^ cells/h	Up to 4.7-fold improved residual activity	(Beneyton et al. 2017)
10	*Candida antarctica* lipase A (Cal-A)	Automated library construction workflow	735 clones	88 clones showed improved substrate discrimination	(Quaglia et al. 2017)
11	*Archaeoglobus fulgidus* esterase (AFEST)	Automated dual-channel microfluidic screening platform	~ 5 × 10⁶ clones	Enantioselectivity increased 700-fold	(Ma et al. 2018)
12	*Staphylococcus aureus* sortase A (Sa-SrtA)	SortEvolve automated screening workflow	~ 480 clones	Catalytic efficiency increased 22-fold	(Zou et al. 2018)
13	*Aspergillus niger* glucose oxidase (GOx)	Automated droplet microfluidic screening	2,000 droplets/s	*k*_cat_ increased 2.1-fold (mutant M6)	(Prodanović et al. 2020)
14	*Sulfolobus acidocaldarius *geranylgeranyl reductase (SaGGR)	Automated TLC separation and detection; Automated acoustic droplet ejection spotting	5-10 × 96-well plates/day	4-fold activity difference distinguishable	(Garabedian et al. 2020)
15	*Streptomyces noursei *cyclodipeptide synthase(AlbC)	Automated library construction; Automated mass spectrometry screening	~4,500 clones (5 s/sample)	F186L mutant producing novel product	(Zhang et al. 2022)

*FACS *Fluorescence-Activated Cell Sorting, *HTS *High-Throughput Screening, *TLC *Thin-Layer Chromatography

In the standalone automation stage (2000s), automated equipment was primarily used to replace specific manual operations in the enzyme engineering workflow, achieving an initial transition from “manual bench work” to “mechanized operation”. A representative study from 2006 on the directed evolution of sialyltransferase (ST) employed FACS to achieve ultra-high-throughput screening of mutant libraries (Aharoni et al. 2006). In this work, researchers constructed an ST mutant library containing over 10⁶ variants and used the FACS system to efficiently screen the entire library in less than two hours. After three rounds of iterative sorting, they successfully identified a variant carrying a single F91Y point mutation, which exhibited up to ~ 367-fold improvement in catalytic efficiency toward certain fluorescently labeled substrates (e.g., bodipy-galactose) compared to the wild-type enzyme. This case also highlights that FACS screening is most effective when product formation can be converted into a retained intracellular fluorescence signal; however, the F91Y improvement was largely bodipy-dependent, limiting its direct transferability to unlabeled or differently labeled substrates. This application demonstrated the substantial potential of FACS as a standalone screening tool in overcoming specific screening throughput bottlenecks. Although upstream library construction and downstream validation steps still required manual intervention, this work laid critical technical groundwork for subsequent more complex integrated systems.

Entering the cascade automation stage (2010s), researchers began to logically connect multiple standalone devices using robotic arms or unified software protocols, achieving automated handover of multiple consecutive steps in the enzyme engineering workflow and significantly improving overall operational efficiency. For example, a 2016 platform integrated key instruments via a central robotic arm to achieve a cascade-automated workflow covering colony picking, cultivation, induction, lysis, and activity measurement, though library construction was still performed manually (Dörr et al. 2016). In the directed evolution of cyclohexanone monooxygenase (CHMO), the researchers used this platform to screen approximately 4,200 mutants and successfully identified a CHMO variant with 2-fold higher resistance against 600 mM product (ε-caprolactone). This case demonstrated that cascade automation, by bridging automation islands, can significantly enhance the throughput and reliability of enzyme engineering experiments. The success of this workflow was attributable not only to increased screening scale, but also to reduced well-to-well variability through standardized experimental procedures. This case highlights that the main value of cascade automation lies in improving reproducibility and data reliability in complex screening workflows, rather than simply increasing throughput.

In the highly automated build–test stage (2020s to present), researchers have focused on integrating the entire core workflow of enzyme engineering, ranging from library construction to activity screening, into a fully equipment-integrated system capable of unattended operation, thereby achieving deep automated coupling of the “build–test” cycle. For instance, in a 2022 study on engineering cyclodipeptide synthase (CDPS), researchers employed an integrated biofoundry to robotically automate mutant library construction, colony picking, microplate cultivation, induced expression, product extraction, and MALDI target plate spotting, followed by high-throughput MALDI-ToF MS analysis, thereby realizing a highly automated workflow from library construction to screening detection (Zhang et al. 2022). Using this system, the researchers successfully screened approximately 4,500 clones and identified the F186L mutant capable of synthesizing the novel cyclodipeptide cFV. This example highlights the main advantage of automation-driven enzyme engineering: it can substantially accelerate repetitive experimental steps and improve workflow standardization, making large-scale screening of enzyme variants more practical. It should be noted that in this workflow, the transfer of MALDI target plates from the preparation area to the mass spectrometer still required manual manipulation, and fully unattended end-to-end operation had not yet been achieved. Thus, the benefits of automation can be further enhanced when downstream analytical instruments are directly integrated into the automated workflow.

### Integrated AI and automation in enzyme engineering

Following the six-level autonomy framework (Levels 0–5), the current integration of AI and automation in enzyme engineering can be broadly categorized into lower-autonomy stage (Levels 1–2) and conditional-to-high autonomy stage (Levels 3–4). Table 3 summarizes representative cases since 2020 of integrated AI-automation systems in enzyme engineering.

**Table 3:** Representative cases of integrated AI and automation in enzyme engineering

No.	Enzyme	AI tools or algorithms	Automated process	Enzyme engineering result	Reference
1	Streptavidin-based artificial metalloenzyme	SVM, XGBoost regression	Automated liquid handling (Tecan EVO 200)	Up to 15-fold higher deallylation activity	(Vornholt et al. 2021)
2	*Pseudomonas aeruginosa* rhamnolipid synthase (RhlA)	BO-EVO	Automated library construction and screening	4.8-fold higher relative yield of rhamnolipid (Rha-C8-C10)	(Hu et al. 2023)
3	GH1 family glycoside hydrolase chimera	Gaussian process-based Bayesian optimization agent	Automated gene assembly, protein expression, thermostability assay	12 °C higher thermostability (T_50_)	(Rapp et al. 2024)
4	*Yersinia mollaretii* phytase (YmPhytase)	ESM-2, EVmutation; low-N supervised regression	iBioFAB platform (7 automated modules)	26.3-fold higher specific activity at pH 6.6	(Singh et al. 2025)
5	*Methanocaldococcus jannaschii* p-Cyanophenylalanyl-tRNA synthetase (pCNF-RS)	ESM-2; ITC sampling; multilayer perceptron	iBioFoundry platform (workstation, sealer/peeler, centrifuge, etc.)	2.4-fold higher incorporation activity for non-canonical amino acid (pAcF)	(Zhang et al. 2025a)
6	*Marinactinospora thermotolerans* amide synthase (McbA)	Augmented ridge regression; Georgiev encoding; EVmutation	Automated liquid handling, DNA library construction	42-fold higher catalytic efficiency for moclobemide synthesis	(Landwehr et al. 2025)
7	Thermophilic archaea Family B DNA polymerase	ESM-2; EVOLVEpro; Chai-1 folding	Automated liquid handling, colony picking, cultivation, centrifugation, etc.	37% lower sequencing error rate (variant DP1)	(Zhang et al. 2026a)
8	*Pseudomonas putida* glycolaldehyde synthase (GALS)	LabScriptAI	Tecan Fluent automated liquid handling, colony picking, cultivation, etc.	3.2-fold (GALD) and 7.0-fold (DHA) higher catalytic efficiency	(Gao et al. 2025)

*SVM *Support Vector Machine, *BO-EVO *Bayesian Optimization-guided Evolutionary Algorithm, *ITC *Information Transport Complexity, *GH1 *glycoside hydrolase family 1, *GALD *glycolaldehyde, *DHA *dihydroxyacetone

Lower-autonomy stage (Levels 1–2): preliminary integration of AI design and automated execution. The hallmark of this stage is the coupling of ML models with automated liquid handling workstations to achieve partial automation of the “design–build–test” cycle, while steps such as library construction, data uploading, or model iteration still require manual intervention. A representative example is the BO-EVO algorithm developed by Hu et al., which integrates Bayesian optimization with evolutionary algorithms and couples them with an automated robotic experimental platform to engineer the rhamnolipid synthesis enzyme (RhlA) from *Pseudomonas aeruginosa* (Hu et al. 2023). Unlike MLDE, CLADE, and related approaches that depend on structural or evolutionary prior information, BO-EVO requires no prior protein knowledge, achieves high computational efficiency, and integrates naturally with high-throughput robotic experimentation. By balancing exploration and exploitation, the framework helps avoid local optima and enables efficient navigation of large sequence spaces. Through four rounds of iterative screening, each involving automated construction and screening of 384 mutants, and after sampling less than 1% of the theoretical sequence space (a total of 160,000 possibilities), the study successfully obtained the AACA quadruple mutant, which showed a 4.8-fold increase in the relative yield of the target product Rha-(C₈–C₁₀) compared to the wild-type enzyme. This study illustrates how early AI–automation integration can substantially accelerate enzyme optimization while still relying on human supervision for key workflow components.

Conditional-to-high autonomy stage (Levels 3–4): deep integration of AI-driven and automated experimentation. This stage achieves highly automated operation of the complete DBTL cycle, where AI models directly guide automated equipment for iterative experimentation with substantially reduced human intervention (Chen et al. 2025; Heo et al. 2025). The work by Zhao’s team represents a benchmark example of deeply integrating traditional PLMs with an automated biofoundry (Singh et al. 2025). The strong performance of this platform likely reflects the integration of complementary sequence-level priors, including ESM-2 and EVmutation, with low-sample supervised learning and a robust closed-loop iBioFAB workflow, enabling informative mutant-library design while partly accounting for epistasis and experimental noise. By embedding ESM-2 and the EVmutation algorithm into seven automated modules of iBioFAB, and screening fewer than 500 variants over four iterative rounds, the team achieved a 16-fold improvement in the ethyltransferase activity of a halogen methyltransferase (AtHMT) and a 26.3-fold increase in the specific activity of a phytase (YmPhytase). Compared with earlier automated enzyme-engineering workflows that often relied on single predictive models, less generalizable expression or assay systems, and limited treatment of epistasis and assay variability, this case better illustrates when AI-guided automation is effective: when model design, assay robustness, and automated DBTL execution are jointly optimized. However, this platform falls within the transition from Level 3 (conditional autonomy) to Level 4 (high autonomy), with only minimal human intervention remaining.

More recently, the AI-Native autonomous biofoundry reported by Zhang et al. represents a recent step toward higher autonomy in enzyme-engineering workflows (Zhang et al. 2026a). Based on a “cloud–edge synergistic” architecture and featuring deep integration of LLMs with the Model Context Protocol (MCP), this system enables AI agents to directly “perceive” instrument status and “actuate” physical workflows, achieving end-to-end automatic generation of executable protocols from natural language instructions across platforms (e.g., MGISP-Smart8), while conventional approaches underperform due to rigid, device-specific scripts that lack the flexibility to handle complex, evolving scientific tasks. In an evolution task targeting a Family B DNA polymerase for use with CoolMPS sequencing, the platform identified an optimal variant that reduced the sequencing error rate by 37% compared with a commercial reference enzyme after three autonomous rounds. The progressively increasing hit rates across the three rounds (~ 60% to 65% to 70%) suggest that the workflow may help mitigate the diminishing returns commonly encountered in iterative directed evolution. This case represents an important step toward the long-term vision of “lights-out” biofoundries and provides a potentially scalable, hardware-agnostic framework for constructing more autonomous enzyme-engineering systems.

Currently, the field is rapidly advancing toward higher-level autonomous closed-loop systems (Chen et al. 2025; Gao et al. 2025). LLM-based multi-agent frameworks, such as LabScriptAI, have begun to enable the automatic generation and self-correction of executable scripts from natural language instructions across multiple automation platforms, including Opentrons, Tecan, and Hamilton (Gao et al. 2025). These advances may help reduce the semantic and software barriers among heterogeneous automation platforms and support the development of more autonomous, human-supervised closed-loop enzyme-engineering workflows. By integrating domain-specific knowledge retrieval, hierarchical task planning, targeted code refactoring, iterative simulator-based validation, and safety controls, LabScriptAI may further improve hardware portability, streamline debugging, and lower the risk of hallucination-prone workflow errors compared with generic LLMs or platform-specific scripting tools.

## Challenges and perspectives of AI and automation in enzyme engineering

Although AI and automation have significantly accelerated enzyme engineering, important scientific and engineering bottlenecks still limit the realization of robust and truly autonomous workflows. These bottlenecks arise not only from model accuracy, but also from data availability, assay compatibility, experimental throughput, workflow integration, and the degree of autonomous decision-making. Therefore, a critical comparison of different AI-enabled paradigms is needed before discussing their specific challenges and future directions.

Table 4 summarizes the operational boundaries of four major paradigms in AI-enabled enzyme engineering: zero-shot PLM prediction, supervised learning, active learning, and AI-biofoundry closed loops. These approaches differ substantially in their dependence on target-specific data, compatible assay formats, expected transferability, and dominant failure modes. In general, zero-shot PLMs are most useful for data-scarce mutation prioritization, supervised and active-learning methods become more powerful when reliable experimental feedback is available, and AI-biofoundry closed loops provide the most promising route toward autonomous optimization but remain constrained by assay automation, infrastructure requirements, and interoperability. This comparison provides a framework for the following discussion, which is organized into two major dimensions: AI-guided enzyme engineering and autonomous enzyme engineering.

**Table 4:** Comparative assessment of major AI and AI-automation paradigms in enzyme engineering

Paradigm	Data needs	Assay compatibility	Transferability	Primary limitations	Reference
Zero-shot PLM prediction	No target-enzyme fitness data required; relies on large-scale unlabeled sequence pretraining and, in some cases, homologous sequence context.	Compatible with low-throughput or expensive assays because only a small prioritized variant set needs validation.	Moderate to broad for mutation prioritization within evolutionarily plausible sequence space; weaker for substrate-specific catalysis and mechanistically novel functions.	Captures evolutionary plausibility rather than catalytic mechanism; limited sensitivity to active-site geometry, substrates, reaction conditions, and higher-order epistasis.	(Hie et al. 2021; Meier et al. 2021; Liu et al. 2025)
Supervised learning	Requires experimentally measured sequence–fitness data from the target enzyme, close homologs, or focused mutant libraries; data quality and training-set design are critical.	Best suited to assays that can generate tens to thousands of quantitative, reproducible measurements; assay consistency strongly affects model reliability.	High within the sampled sequence–function landscape but generally limited under out-of-distribution shifts, new substrates, or different assay formats.	Data hungry; vulnerable to assay noise, benchmark leakage, sparse “hole-filled” landscapes, and poor extrapolation beyond the training distribution.	(Mazurenko et al. 2019; Wittmann et al. 2021; Wu et al. 2019; Yang et al. 2019)
Active learning / Bayesian optimization	Requires an initial experimental dataset followed by iterative acquisition of new sequence–fitness data; most effective when uncertainty estimates are informative.	Requires assays that are sufficiently robust and repeatable for multiple design–build–test rounds; compatible with moderate-throughput screening.	Moderate; can adapt to the target landscape during optimization, but transferability across enzymes is usually limited unless representations or priors are reused.	Performance depends on model calibration, exploration–exploitation balance, assay noise, and landscape ruggedness; build–test latency can limit iteration speed.	(Biswas et al. 2021; Yang et al. 2025a; Yu et al. 2023b)
AI-biofoundry closed loops	Requires continuous generation, capture, and integration of standardized DBTL data; benefits from both prior models and newly generated experimental data.	Most compatible with high-throughput, automation-ready assays and standardized build–test workflows; difficult for low-throughput or manually intensive analytical assays.	Potentially broad at the workflow level, but practical transferability depends on hardware interoperability, assay portability, data standards, and biological context.	High infrastructure cost; limited accessibility; interoperability barriers; incomplete autonomy in fault detection, replanning, and strategic decision-making.	(Carbonell et al. 2018; Chen et al. 2025; MacLeod et al. 2020; Zhang et al. 2026b)

### AI-guided enzyme engineering: from prediction to mechanistic understanding

#### Data limitations and benchmark reliability

Challenge: The performance of AI models in enzyme engineering is fundamentally constrained by data quality issues that extend beyond simple data scarcity. These limitations primarily arise from several interconnected factors, including (a) dataset imbalance and negative-data scarcity, (b) assay heterogeneity and incomplete metadata, and (c) benchmark leakage.

Experimental datasets are heavily skewed toward well-studied enzyme families, particularly oxidoreductases, transferases and hydrolases, which dominate many current public enzyme datasets, while negative results are rarely reported (Malli et al. 2025; Moorhoff et al. 2025; Prešern and Goličnik 2023). This positive-only bias, together with assay heterogeneity and incomplete metadata, hinders meaningful data integration and cross-study comparison, as inconsistencies in database annotations and missing experimental details reduce data quality and comparability. The scarcity of negative and out-of-distribution (OOD) samples further compromises model generalization to understudied enzymes or non-natural substrates (Moorhoff et al. 2025; Yang et al. 2024).

More critically, benchmark leakage has led to inflated performance estimates that do not reflect true generalization. Re-evaluations of supervised kinetic predictors under strict sequence-based splits showed that performance drops sharply on low-homology test proteins, highlighting how train–test similarity can substantially overestimate model capability (Kroll A and Lercher MJ 2024). Similar leakage has also been documented across protein-protein interaction, binding-affinity, and mutation-effect benchmarks (Bernett et al. 2024; Graber et al. 2025; Kapoor and Narayanan 2023).

Perspective: Addressing these challenges will require advances in three areas: (a) balanced fitness-landscape generation, (b) standardized metadata infrastructure, and (c) leakage-aware benchmark design. Systematic generation of balanced fitness landscapes via deep mutational scanning, supported by high-throughput microfluidic screening platforms, can produce comprehensive sequence-fitness maps containing both positive and negative variants (Mazurenko et al. 2019; Vasina et al. 2023). Adoption of FAIR principles, STRENDA guidelines, and standardized formats such as EnzymeML ensures machine-readable and complete metadata, while community-curated repositories such as the Open Enzyme Database (OED) demonstrate the value of integrating validated kinetic parameters into AI-ready formats (Malzacher et al. 2024; Pleiss 2021; Range et al. 2021; Yuan et al. 2026). Furthermore, leakage-aware benchmark design is essential for realistic model evaluation. Sequence-cluster-based splits (e.g., clustering at < 40% sequence identity) and temporal partitions should replace random splitting to avoid homolog contamination and inflated performance estimates (Malli et al. 2025; Wang et al. 2025a, b). Structure-based filtering, as exemplified by PDBbind CleanSplit, eliminates train-test structural similarities and reveals true model performance (Graber et al. 2025). Ultimately, overcoming data limitations requires a paradigm shift from convenience-based data collection to systematic, standardized, FAIR-compliant data generation, enabling reliable model training and evaluation.

#### Limited generalization under out-of-distribution shifts

Challenge: Even with high-quality training data, AI models frequently fail to generalize beyond their training distribution. Such OOD failures are particularly evident across several scenarios, including (a) positional extrapolation, (b) protein-family distribution shift, (c) evolutionary or species bias in PLMs, and (d) mutation-order extrapolation in the presence of epistasis (Ding and Steinhardt 2024; Freschlin et al. 2024; Michael et al. 2024).

Models trained on in-distribution samples often show sharply degraded performance when evaluated on unseen sequence families, distant homologs, or novel mutation combinations. Sequence-only PLMs may generalize better than supervised predictors under certain distribution shifts, but their predictions remain strongly shaped by evolutionary priors rather than explicit mechanistic understanding (Zeng et al. 2026). Performance further declines when models trained on low-order (single and double) mutations are extrapolated to combinatorial mutation spaces with strong epistasis, where higher-order residue interactions become increasingly important (Freschlin et al. 2024; Michael et al. 2024). These observations suggest that current AI models remain far more reliable for interpolation within known sequence manifolds than for true extrapolative design.

Perspective: Improving generalization will require progress across multiple directions, including (a) rigorous OOD benchmark design, (b) prospective wet-lab validation, (c) transfer learning for low-data systems, and (d) physics-informed modeling. Task-specific splits, such as position-level, mutation-degree, chronological, or homology-aware clustering, should be selected according to the intended extrapolation setting (Michael et al. 2024). Prospective wet-lab validation remains the gold standard, as retrospective benchmarks with leakage often fail to predict real-world performance (Zeng et al. 2026). For understudied systems, meta-transfer learning and few-shot fine-tuning of PLMs can improve generalization by training on auxiliary tasks from related proteins and incorporating MSA-derived pseudo-labels (Zhou et al. 2024b). Finally, integrating physics-based features such as Rosetta energy terms, residue flexibility, and active-site geometry into neural network inputs may improve extrapolation to unseen mutations, particularly for structurally characterized proteins (Gelman et al. 2025).

#### Mechanistic interpretability in AI-driven enzyme design

Challenge: A central unresolved question in AI-guided enzyme engineering is whether current models genuinely learn catalytic mechanisms or primarily exploit evolutionary sequence statistics. In other words, sequence likelihood does not necessarily translate into functional fitness (Gordon et al. 2025; Kolchina et al. 2026). This interpretability gap arises from the strong dependence of current models on evolutionary priors and their limited ability to explicitly represent catalytic determinants.

A fundamental limitation is that evolutionary fitness and catalytic fitness are related but not equivalent. Protein sequences reflect historical evolutionary selection rather than direct optimization of catalytic performance (Starr and Thornton 2016; Bloom et al., 2006), whereas enzyme catalysis is governed by physicochemical processes operating at the atomic level (Warshel et al., 2006). Sequence-based PLMs primarily infer mutational effects from sequence conservation and co-evolution (Rives et al., 2021; Meier et al. 2021), but catalytic efficiency depends on factors such as transition-state stabilization, electrostatic preorganization, substrate positioning, proton-transfer pathways, and conformational dynamics (Warshel et al., 2006; Fried and Boxer, 2017; Kamerlin and Warshel, 2010). Consequently, models may successfully capture evolutionary regularities without necessarily learning the mechanistic basis of catalysis, particularly for non-natural substrates, novel reaction chemistries, or mutations outside evolutionary sequence space (Shao et al. 2025).

Moreover, apparent interpretability may simply reflect advanced pattern recognition rather than genuine mechanistic reasoning. Attention maps, saliency analyses, and co-folding models can identify contact patterns or active-site residues, but these signals do not necessarily imply causal understanding of enzyme function. Recent studies have further shown that high-confidence structural predictions may still contain physically implausible ligand poses or steric conflicts under distribution shifts (Masters et al. 2025). Collectively, these observations suggest that current AI systems are better viewed as tools for mutation prioritization and search-space reduction than as mechanistic models capable of explaining enzyme catalysis.

Perspective: Bridging this gap will require a shift from statistical prediction toward hybrid mechanistic AI, moving from prediction to explanation and ultimately to causal understanding. Several advances are particularly important. (a) Integrating physics-based priors into AI models is essential. Combining PLMs with biophysical descriptors, such as Rosetta energy terms, active-site geometry, residue flexibility, or MD-derived conformational ensembles, can constrain predictions with physically meaningful information and improve extrapolation to unseen mutations (Gelman et al. 2025). (b) Physics-based simulations should complement AI predictions to provide mechanistic explanations. Molecular dynamics, docking, and QM/MM simulations can reveal how mutations alter electrostatic environments, transition-state stabilization, substrate positioning, or conformational dynamics. Such hybrid workflows are particularly valuable for explaining distal or epistatic mutations that sequence-only models cannot mechanistically rationalize (Shao et al. 2025). (c) Structure-aware and multimodal foundation models represent a promising direction. Models integrating sequence, structure, and dynamics, such as graph neural networks, structure-aware PLMs, and multimodal generative architectures, may better capture nonlocal interactions and catalytic constraints than sequence-only models. Future benchmarks should therefore evaluate not only predictive accuracy but also whether models recover known catalytic principles, including electrostatic preorganization, transition-state stabilization, and allosteric coupling. Only through such mechanistically grounded evaluation can the field distinguish genuine catalytic understanding from sophisticated statistical pattern matching.

#### Epistasis-driven ruggedness in multi-mutation landscapes

Challenge: The transition from single- to multi-mutation engineering is substantially complicated by epistasis, defined as the non-independence of mutational effects, which makes multi-mutant phenotypes often difficult to predict from single-mutant measurements alone. As the number of targeted mutations increases, the combinatorial sequence space expands exponentially, while epistatic interactions render exhaustive experimental characterization impractical. This challenge is particularly driven by several forms of epistasis, including (a) sign epistasis, (b) reciprocal sign epistasis, and (c) higher-order epistasis.

Sign epistasis occurs when a mutation is beneficial in one genetic background but deleterious in another, creating context-dependent fitness effects that cannot be reliably inferred from single-mutant measurements alone (Starr and Thornton 2016). Reciprocal sign epistasis can generate multiple local fitness optima, increasing landscape ruggedness and constraining accessible evolutionary trajectories (Poelwijk et al. 2019). Higher-order epistasis involving three-way, four-way, or even more complex interactions further increases landscape complexity by making even pairwise mutational effects strongly context-dependent. Experimental fitness landscape studies suggest that higher-order interactions can contribute substantially to observed non-additive effects, although their magnitude varies across proteins and mutational contexts (Sethi and Zhou 2025).

These effects create highly rugged fitness landscapes in which additive models often fail to accurately estimate multi-mutant fitness, sometimes predicting highly active variants that prove experimentally nonfunctional (Sethi and Zhou 2025). In enzyme engineering, this challenge is particularly pronounced because catalytic performance often depends on coordinated interactions among active-site residues, second-shell residues, and distal allosteric positions that collectively regulate substrate binding, transition-state stabilization, electrostatic preorganization, and conformational dynamics. Consequently, models that neglect higher-order interactions often show limited predictive reliability in multi-mutation design, particularly for structurally or functionally coupled residues.

Perspective: Addressing the epistasis-combinatorial challenge requires strategies that explicitly model, learn, or efficiently navigate rugged fitness landscapes without exhaustive enumeration. Several complementary approaches are showing strong potential. (a) Active learning enables iterative exploration guided by model uncertainty, allowing experiments to prioritize the most informative variants while balancing exploration and exploitation. By progressively updating surrogate models with newly acquired data, active learning can substantially improve sampling efficiency in protein engineering (Jiang et al. 2024a, b). (b) Bayesian optimization (BO) provides a related sequential optimization framework that leverages probabilistic surrogate models with uncertainty estimates to identify promising multi-mutants while minimizing experimental burden. In a representative study, BO-EVO (a hybrid of Bayesian optimization and evolutionary algorithms) identified near-optimal variants after screening only a small fraction of the theoretical sequence space (Hu et al. 2023). (c) Epistasis-informed combinatorial library design can improve sampling efficiency by prioritizing functionally or structurally coupled residues. While direct coupling analysis (DCA) does not directly measure epistasis, it infers evolutionary couplings from sequence alignments, providing a basis for focused library construction with reduced screening burden (Marks et al. 2011; Morcos et al. 2011). (d) DL models, including transformer-based architectures, offer scalable computational frameworks for modeling higher-order residue interactions. By incorporating residue–residue interactions, these models are better suited than additive or independent-site sequence models for capturing complex, including higher-order, epistatic dependencies (Sethi and Zhou 2025).

Although epistasis remains a central bottleneck in multi-mutation engineering, explicit modeling of fitness landscape ruggedness can substantially improve search efficiency and predictive performance. Future progress will likely depend on deeper integration of active learning, structure-aware AI, and intelligent combinatorial design, together with benchmarking datasets containing comprehensive multi-mutant fitness measurements for rigorous evaluation of epistasis-modeling strategies.

#### Integrating sequence, structure, and generative AI paradigms

Challenge: Current AI frameworks in enzyme engineering remain largely sequence-centric, with PLMs serving as the dominant paradigm for mutation-effect prediction. Although PLMs have demonstrated strong zero-shot performance by learning evolutionary priors from large sequence corpora (Cheng et al. 2024), their limitations are increasingly evident in several aspects, including (a) lack of explicit structural representation, (b) insufficient modeling of long-range coupling and dynamics, and (c) limited capability for de novo design.

Sequence-only models lack explicit representation of three-dimensional active-site geometry, substrate-binding interactions, and catalytic residue organization, all of which are critical for enzyme function (Garcia-Vinuesa et al. 2025). They also struggle to capture long-range structural coupling, allosteric communication, and conformational dynamics that are not fully encoded in sequence statistics alone (Huynh et al. 2025). Moreover, PLMs are primarily optimized for mutation scoring or ranking rather than de novo design of entirely new folds or catalytic sites (Winnifrith et al. 2024). These limitations highlight the incomplete nature of sequence-centric modeling and have driven increasing interest in structure-aware DL and generative AI. However, the complementary roles of these paradigms within enzyme engineering remain insufficiently defined.

Perspective: Future progress will likely depend on integrating sequence-based, structure-aware, and generative AI into unified workflows rather than treating them as competing paradigms (Shi et al. 2026b). Alignment-based sequence models are effective for extracting evolutionary constraints within known families, while large-scale PLMs capture broad evolutionary priors across diverse sequences (Paul et al. 2023). Structure-aware models, including geometric DL and graph neural networks, complement PLMs by explicitly modeling active-site geometry, residue interactions, and long-range structural dependencies, thereby improving prediction for low-homology proteins and epistatic mutations (Cheng et al. 2024; Fei et al. 2025). Meanwhile, generative AI, particularly diffusion and inverse-folding models, expands exploration beyond natural sequence space and enables de novo scaffold and active-site design (Ahern et al. 2025; Watson et al. 2023). The most promising direction is therefore multimodal foundation models that jointly learn sequence, structure, and dynamics, enabling integrated DBTL workflows that combine evolutionary priors, physical constraints, and generative design capability (Zhou et al. 2024a).

#### Multi-objective optimization under industrial constraints

Challenge: Industrial applications demand enzymes with simultaneously optimized properties, including activity, thermostability, selectivity, expression yield, and solvent tolerance (Khan 2025; Victorino da Silva Amatto et al. 2021). These challenges primarily stem from several interconnected factors, including inherent biophysical trade-offs, the limited multi-objective capability of current AI models, and the scarcity of high-quality multi-property datasets.

At the biophysical level, a central trade-off is that stabilizing mutations may compromise catalytic flexibility, as the conformational dynamics required for activity can be restricted by enhanced rigidity (Hou 2023; Khan 2025). For example, structure-based design improved the thermostability of xylanase and zearalenone hydrolase but caused severe or even complete loss of catalytic activity (Wu et al. 2026). At the modeling level, most current AI models remain optimized for single-property prediction and often fail to capture trade-offs among coupled traits. Meanwhile, the high experimental cost of multi-property characterization further restricts the availability of training data for multi-objective models (Ndochinwa et al. 2024).

Perspective: Addressing multi-objective optimization requires a shift from single-property prediction toward integrated frameworks that explicitly model trade-offs and identify Pareto-optimal solutions. In the near term, one practical strategy is to combine orthogonal single-property predictors, such as thermostability, solubility, and activity estimators (Liu et al. 2025), and rank variants using intersection- or Pareto-based criteria (Luo et al. 2025). Beyond this modular strategy, another promising direction is the development of multi-task and multimodal models that jointly predict multiple enzyme properties from unified representations, thereby enabling the model to capture biophysical couplings among interdependent traits that single-property models often overlook. EZPro-Multi exemplifies this trend by integrating protein and substrate embeddings to simultaneously predict *k*_cat_, ΔΔG, and ΔSol, improving hit rates compared with single-property prediction (Sui et al. 2026). At the experimental level, active learning and automated high-throughput platforms can improve sampling efficiency, generate multi-property datasets, and accelerate validation. This is particularly the case for biofoundry-style systems that measure multiple properties from the same variant library. Ultimately, robust industrial enzyme engineering will require tightly integrated AI-automation systems capable of jointly optimizing multiple performance constraints under realistic deployment conditions.

### Autonomous enzyme engineering: from automation islands to closed-loop intelligence

Automation has progressively transformed enzyme engineering from low-throughput, manually executed, and sequential experimentation toward increasingly high-throughput, parallelized, and data-driven DBTL workflows. Rather than operating as isolated automation islands, emerging automated platforms are beginning to connect design, build, test, and learning into iterative closed-loop optimization cycles. However, substantial technical and infrastructural barriers still hinder the widespread implementation of such autonomous systems.

#### Accessibility and interoperability barriers in laboratory automation

Challenge: Despite the transformative potential of laboratory automation in enzyme engineering, access remains highly uneven due to a persistent divide between low-cost open platforms and high-end integrated biofoundries. These barriers primarily arise from several interconnected factors, including limited accessibility to advanced infrastructure and poor interoperability among heterogeneous laboratory instruments.

Low-cost systems such as open-source liquid handlers substantially improve accessibility and enable automation of routine tasks, but they often provide more limited throughput, device integration, and validated performance ranges than highly integrated commercial platforms. As a result, they are well suited for partial workflow automation but often struggle to support robust end-to-end DBTL execution. In contrast, high-end biofoundries provide highly precise, scalable, and tightly integrated automation with superior reproducibility, but their high capital cost, maintenance requirements, and need for specialized personnel place them beyond the reach of most academic laboratories. This creates a fundamental trade-off between accessibility and performance: affordable systems improve adoption but may lead to reduced reproducibility, whereas fully integrated systems maximize reproducibility but limit broad accessibility (Holland and Davies 2020; Stephenson et al. 2023). In practice, reduced reproducibility in low-cost platforms often arises from less standardized liquid handling, less robust calibration, and greater dependence on manual intervention between workflow steps. This reproducibility advantage in high-end systems largely stems from tightly controlled hardware synchronization, automated quality control, and standardized software orchestration across the entire DBTL cycle. Interoperability represents an additional major barrier. Many laboratory instruments, including liquid handlers, thermocyclers, plate readers, and colony pickers, still operate as isolated automation islands using proprietary software, communication protocols, and data formats. Consequently, integrating heterogeneous instruments into unified closed-loop workflows often requires custom orchestration software and substantial engineering effort, creating a major bottleneck for scalable autonomous experimentation (Zhang et al. 2025c).

Perspective: Bridging the accessibility and interoperability gap will require coordinated advances in modular automation, shared infrastructure, and standardized communication. Modular automation offers a practical middle ground, allowing laboratories to automate key bottlenecks such as PCR setup, liquid transfer, or activity screening without building fully autonomous facilities. Shared biofoundries and cloud laboratories can further democratize access to advanced automation by distributing infrastructure costs across users (Holowko et al. 2020; Tellechea-Luzardo et al. 2022).

Equally important is the adoption of vendor-neutral communication standards such as SiLA2 and standardized data frameworks such as AnIML or EnzymeML, which can reduce integration complexity and enable plug-and-play interoperability across instruments (Juchli 2022; Malzacher et al. 2024). Looking ahead, digital twin technologies may further accelerate deployment by enabling virtual workflow validation and resource scheduling before physical execution. Together, these developments can shift laboratory automation from isolated devices toward interoperable and scalable systems, making autonomous enzyme engineering more reproducible, accessible, and practical across laboratories with diverse resource levels.

#### Build-test bottlenecks in closed-loop enzyme engineering

Challenge: Even when AI models prioritize promising multi-site variants, experimental construction and validation remain major rate-limiting steps in closed-loop enzyme engineering due to a persistent mismatch between rapid in silico design and slower wet-lab implementation. These bottlenecks are particularly evident in both the build and test stages.

In the build stage, bottlenecks arise from limited efficiency in multi-site combinatorial mutagenesis and library construction. Generating multi-mutant variants remains resource-intensive, as PCR-based approaches often require iterative cloning, while commercial gene synthesis remains costly for large libraries (Hoose et al. 2023). In addition, library size scales exponentially with mutation number, leading to uneven coverage and accumulation of assembly errors that reduce effective exploration of sequence space (Gali et al. 2024). Expression constraints further restrict throughput, particularly for heterologous and eukaryotic enzymes, where folding inefficiency, toxicity, or lack of post-translational modifications can significantly limit functional protein yield.

In the test stage, the dominant limitation is throughput asymmetry between AI-driven design and experimental characterization. While computational methods can rapidly generate large variant sets, experimental screening often remains orders of magnitude slower, especially for chromatographic or mass spectrometry-based assays (Bunzel et al. 2018; Markel et al. 2020). Although microplate-based assays enable moderate throughput, many industrially relevant reactions still lack suitable high-throughput readouts. Emerging ultrahigh-throughput technologies, such as droplet-based microfluidics, substantially increase screening capacity, but their cost and operational complexity limit broad accessibility (Markel et al. 2020). Consequently, experimental validation remains the primary bottleneck for iterative closed-loop optimization.

Perspective: Addressing build-test bottlenecks will require coordinated advances in molecular construction, expression systems, and screening technologies to better align experimental throughput with AI-driven design scales. For the build stage, modular DNA assembly strategies such as Golden Gate and Gibson Assembly provide efficient routes for rapid construction of combinatorial libraries, particularly when combined with standardized genetic parts (Püllmann et al. 2019; Thomas et al. 2015). Cost-effective oligonucleotide pool synthesis further enables large-scale variant generation without reliance on individual gene synthesis (Hoose et al. 2023). When integrated with liquid-handling automation, these approaches can substantially reduce manual workload and improve reproducibility in library construction workflows.

For the test stage, cell-free protein synthesis (CFPS) offers a key solution by decoupling protein production from cellular constraints, enabling rapid expression directly from linear DNA templates and facilitating faster iteration cycles (Jun et al. 2025; Silverman et al. 2019). This is particularly advantageous for difficult-to-express enzymes where cellular systems impose folding or toxicity constraints. In parallel, continued development and broader democratization of ultrahigh-throughput analytical platforms such as droplet-based microfluidics will be critical for scaling label-free screening capacity (Markel et al. 2020). Overall, integrating automated DNA assembly, cell-free expression systems, and high-throughput or ultrahigh-throughput screening within biofoundry-enabled workflows will be essential to reduce build-test latency and support truly autonomous enzyme engineering systems.

#### Autonomy gap in closed-loop enzyme optimization

Challenge: Despite substantial progress in integrated AI-automation systems, fully autonomous closed-loop enzyme optimization remains unrealized. Current state-of-the-art platforms operate at approximately Level 3–4, where experimental execution is highly automated but decision-making still depends on expert supervision (Beal and Rogers 2020; Singh et al. 2025). Near-term progress will likely focus on achieving more robust Level 4 autonomy through improved adaptive control and fault recovery, whereas Level 5 “lights-out” laboratories remain a long-term goal. Importantly, the remaining autonomy gap does not primarily arise from insufficient robotic execution, as robotic capabilities have improved substantially. Instead, it largely stems from limited autonomous reasoning for supervision, exception handling, and strategic replanning.

This autonomy gap is particularly evident in several areas, including (a) human-dependent initialization and goal specification, (b) limited fault tolerance and recovery, and (c) incomplete adaptive reasoning. Optimization objectives, assay design, screening thresholds, and biological constraints still must be defined by experts before the DBTL cycle begins. Instrument drift, liquid-handling errors, and cross-device failures remain common, yet most systems cannot autonomously detect, diagnose, and recover from such failures without manual intervention (Stephenson et al. 2023). Moreover, although active-learning frameworks can recommend subsequent variants, current systems generally cannot autonomously determine when to modify experimental conditions, redefine search strategies, or terminate unproductive optimization trajectories. Consequently, current closed-loop platforms are better characterized as expert-supervised autonomy rather than fully autonomous laboratories.

Perspective: Closing the autonomy gap requires a transition from workflow automation toward self-correcting and decision-capable autonomous systems. Several advances are particularly important. (a) AI agents for experimental orchestration: LLM-based multi-agent systems increasingly enable protocol generation, workflow coordination, and simulation-based code correction across heterogeneous instruments, providing a foundation for higher-level experimental autonomy (Gao et al. 2025; Zhang et al. 2026a). (b) Real-time anomaly detection and autonomous recovery: integrating instrument telemetry with machine-learning-based fault detection could enable automatic recalibration and error diagnosis without interrupting continuous operation. (c) Hardware-agnostic orchestration layers: standardized interfaces such as SiLA2 and programmable abstraction frameworks can reduce integration complexity and improve system robustness across heterogeneous devices (Wierenga et al. 2023).

In the near term, however, Level 5 full autonomy should be regarded as a long-term goal rather than an imminent reality. A more realistic trajectory is toward human-on-the-loop systems, in which AI manages routine closed-loop optimization while human experts retain oversight of goal definition, biological validation, biosafety, and strategic decision-making (Beal and Rogers 2020). Thus, the future of autonomous enzyme engineering will likely emphasize augmented autonomy rather than complete human replacement.

#### Biosafety, ethics, and governance in autonomous enzyme engineering

Challenge: The convergence of AI and automation in enzyme engineering introduces biosafety and ethical risks that extend beyond conventional laboratory safety concerns. These risks arise from several interconnected aspects, including (a) the dual-use potential of AI-driven enzyme design, (b) the amplification of experimental capability through automation, and (c) the possibility of accidental release or misuse of engineered enzymes.

AI models developed for beneficial enzyme design may also have dual-use potential, as similar capabilities could, in principle, be repurposed to aid the design or optimization of enzymes, proteins, or other biomolecules with hazardous functions (Pannu et al. 2025; Wheeler 2025). Increasing automation further amplifies operational capability: autonomous or cloud-connected biofoundries could reduce the expertise required to execute complex experimental workflows, thereby lowering barriers to misuse if systems are compromised or inadequately controlled (Pannu et al. 2025). In addition, accidental release or misuse of engineered enzymes with enhanced stability or novel catalytic activities may introduce environmental or biosecurity risks. Ethical concerns also arise regarding accountability, namely who bears responsibility when autonomous systems generate unsafe designs or execute harmful protocols. More fundamentally, current governance frameworks, including institutional biosafety oversight and existing biosecurity regulations, were largely designed for conventional wet-lab research and remain insufficient for AI-driven design and autonomous closed-loop experimentation (Bloomfield et al. 2024). As AI models become more capable and automation more accessible, the gap between technological capability and governance readiness may continue to widen.

Perspective: Mitigating these risks requires a layered governance framework integrating technical safeguards, operational controls, and regulatory oversight. Several directions are particularly important. (a) Access control and user verification for high-capability AI design tools and automated facilities can reduce unauthorized use. (b) Safety constraints embedded within AI systems, including sequence screening, capability restriction, and inference-time guardrails, can reduce dual-use risks (Wang et al. 2025a, b). (c) Continuous risk assessment, red-teaming, and adversarial testing of integrated AI-automation platforms will be essential for identifying emerging vulnerabilities before deployment (Pannu et al. 2025).

At the governance level, audit trails and logging can improve traceability across design, synthesis, and testing workflows (Wheeler 2025). International coordination is needed to establish harmonized biosafety standards for autonomous biofoundries and AI-enabled biological design, building on frameworks such as the Biological Weapons Convention and WHO guidance (Bloomfield et al. 2024). Ultimately, responsible autonomous enzyme engineering will require safety, ethics, and governance to be embedded throughout the entire DBTL cycle, ensuring that oversight evolves in parallel with technological progress rather than reacting only after risks emerge.

## Conclusions

AI and laboratory automation are jointly transforming enzyme engineering from empirical screening into data-driven, iterative, and increasingly autonomous optimization. This transformation has already improved mutation prioritization, experimental throughput, and DBTL-cycle efficiency, but its industrial deployment remains constrained by three major gaps: the data gap between vast sequence space and limited high-quality experimental annotations; the mechanism gap between predictive accuracy and causal understanding of enzyme function; and the deployment gap between sophisticated automation systems and broadly accessible laboratory implementation. Addressing these challenges will require advances in both data and infrastructure. Key priorities include FAIR-compliant datasets, leakage-aware benchmarks, hybrid models integrating sequence, structure, dynamics, and biophysical priors, and modular, interoperable automation platforms. In the longer term, autonomous closed-loop platforms combining active learning, robust robotic execution, real-time fault recovery, and human-on-the-loop oversight will be central to next-generation enzyme engineering. The main contribution of this review is a unified and critical framework that connects the evolution of AI, laboratory automation, and their convergence across autonomy levels in enzyme engineering. By combining representative successes with failure modes, persistent limitations, and potential solutions, this review provides both a conceptual reference and a practical roadmap for AI-guided, closed-loop, and autonomous enzyme engineering.

## Supplementary Information

Supplementary Material 1.

## Data Availability

Not applicable.

## Abbreviations

AI: Artificial intelligence
AlphaFold2: AlphaFold version 2
AnIML: Analytical Information Markup Language
ArMs: Artificial metalloenzymes
ASR: Ancestral sequence reconstruction
BO‑EVO: Bayesian Optimization‑guided Evolutionary Algorithm
CDPS: Cyclodipeptide synthase
CFPS: Cell‑free protein synthesis
CHMO: Cyclohexanone monooxygenase
CLADE: Classification‑based active learning‑directed evolution
CNN: Convolutional neural network
CP: Colony picking
DBTL: Design‑build‑test‑learn
DCA: Direct coupling analysis
DHA: Dihydroxyacetone
DL: Deep learning
DLKcat: Deep Learning‑based kcat predictor
DNNs: Deep neural networks
EnzymeML: Enzyme Markup Language
ESM: Evolutionary Scale Modeling
EVmutation: Evolutionary Mutation model
E‑value: Enantiomeric ratio
FACS: Fluorescence‑activated cell sorting
FAIR: Findable, Accessible, Interoperable and Reusable
FFT‑PLSR: Fast Fourier transform‑partial least squares regression
GALD: Glycolaldehyde
GAN: Generative adversarial network
GBA: Global biofoundry alliance
GBRT: Gradient‑boosted regression tree
GH1: Glycoside hydrolase family 1
GP: Gaussian process
HPLC: High performance liquid chromatography
HTS: High‑throughput screening
iCASE: Isothermal Compressibility‑assisted Dynamic Squeezing
ITC: Information transport complexity
KSI: Ketosteroid isomerase
LH: Liquid handling
Linear SVR: Linear support vector regressor
LLMs: Large language models
MALDI‑ToF MS: Matrix‑assisted laser desorption/ionization time‑of‑flight mass spectrometry
MCP: Model context protocol
MD: Molecular dynamics
MLDE: Machine‑learning‑directed evolution
MSA: Multiple sequence alignment
OED: Open Enzyme Database
OOD: Out‑of‑distribution
PCR: Polymerase chain reaction
PDB: Protein Data Bank
PDBbind: Protein Data Bank binding database
PLMs: Protein language models
QM/MM: Quantum mechanics/molecular mechanics
RF: Random forest
RNNs: Recurrent neural networks
Sav: Streptavidin
SiLA2: Standardization in Lab Automation 2
ST: Sialyltransferase
STRENDA: Standards for Reporting Enzymology Data
SVM: Support vector machines
SVR: Support vector regression
TLC: Thin‑layer chromatography
Tm: Melting temperature
VAE: Variational autoencoder
WHO: World Health Organization
XGBoost: Extreme gradient boosting
ΔΔG: Change in Gibbs free energy upon mutation
