# Supplementary figures and images for: Artificial intelligence and automation in enzyme engineering: evolution, advances, and future perspectives
Source: Bioresour Bioprocess. 2026 Jul 10;13(1):101. doi: 10.1186/s40643-026-01096-3 (PMC13354739; doi:10.1186/s40643-026-01096-3)

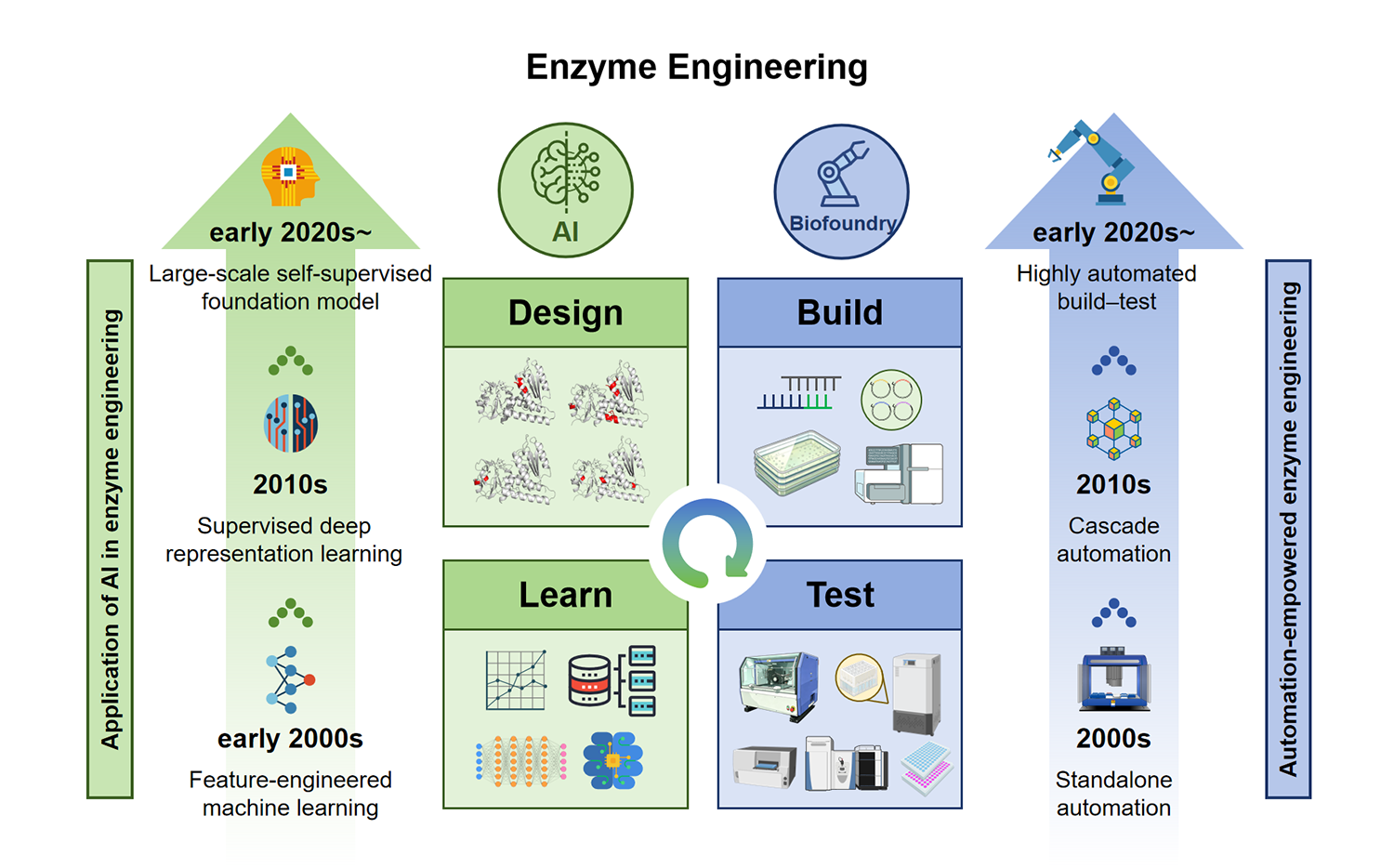

Supplement: Supplementary file 1 — Supplementary Material 1. [file 40643_2026_1096_MOESM1_ESM.tif]
